# Large Language Model–Based Patient Simulation to Foster Communication Skills in Health Care Professionals: User-Centered Development and Usability Study

**DOI:** 10.2196/81271

**Published:** 2025-12-12

**Authors:** Ahmed Elhilali, Andy Suy-Huor Ngo, Daniel Reichenpfader, Kerstin Denecke

**Affiliations:** 1 Institute Patient-centered Digital Health Bern University of Applied Sciences Biel Switzerland

**Keywords:** chatbot, large language model, medical education, patient simulation, vignette

## Abstract

**Background:**

Case-based learning using standardized patients is a key method for teaching communication skills in medicine. Besides logistical and financial hurdles, standardized patients portrayed by actors cannot cover the complete diversity of sociodemographic factors of patients. Large language models (LLMs) show promise for creating scalable patient simulations and could probably cover a broader diversity of factors. They could also be integrated into the continuous training of future health care professionals’ communication and interaction skills.

**Objective:**

This study aimed to introduce the system architecture of a digital tool that leverages LLMs to simulate patient conversations for medical education, focusing specifically on medical history taking. Through an explorative analysis, we aimed to assess the tool’s usability and examine differences between LLMs in simulating patient encounters.

**Methods:**

We followed a user-centered design process, gathering initial requirements from 2 medical students. We then developed a fully functional web prototype using a Python Flask backend and a PostgreSQL database, integrating 5 LLMs from OpenAI, Anthropic, and xAI. The system includes an artificial intelligence–assisted case vignette generator and a dynamic patient simulator. For the explorative analysis of the prototype, we conducted a task-based usability test with 5 medical students, measuring their experience using the System Usability Scale (SUS) questionnaire and qualitative questions. We then conducted an explorative analysis in which 4 practicing physicians evaluated the simulation quality of 3 models (Grok 3, GPT-4, and Claude 3 Opus) across 7 criteria on a 5-point Likert scale.

**Results:**

Usability testing yielded a mean SUS score of 91.5 (SD 8.40), indicating high perceived usability in a small formative sample. Students praised the system’s simplicity and intuitive design but noted the absence of a formal conclusion and performance feedback, expressing a desire for a “didactic loop” to maximize learning. The models showed limitations in simulating uncertainties and memory lapses, responding to follow-up questions, and producing natural conversational flow. They perform well in simulating a coherent symptom profile, in using patient-like language, and in describing a realistic timeline and symptom progression. The differences among the models were not statistically significant. Ratings showed limited discriminative reliability (Kendall W=0-0.19, ie, very low) and a ceiling effect, with most scores clustered at 4-5, constraining interpretation; all group differences should therefore be viewed as exploratory.

**Conclusions:**

We successfully developed a highly usable patient simulation tool that serves as a foundation for further development. Our results show that while the tool could be effective for communication training, its full potential will only be realized by integrating an automated feedback mechanism to create a complete didactic loop, as requested by the test users. Future work should assess in more depth the differences among the models in simulating psychosocial patient characteristics.

## Introduction

### Background

Effective communication between physicians and patients is an established core competency in medical education and practice. The demand for more patient-centered medical decision-making requires that the various needs of patients must be identified in conversations [1]. Nevertheless, current guidelines for doctor-patient communication show deficits in communication skills, especially in dealing with psychosocial factors and cultural diversity, which are increasingly shaping everyday clinical practice [2].

Case-based learning is a well-established pedagogical strategy in medical education, designed to foster students’ clinical reasoning by engaging them with authentic patient scenarios [3]. Unlike didactic teaching methods, case-based learning promotes active, contextualized learning, encouraging students to analyze complex clinical situations and develop vital decision-making skills [4]. A cornerstone of case-based learning—particularly for practicing communication competencies—is the use of standardized patients. Traditionally, these standardized patients are represented by trained actors who take the role of patients with specific medical histories and personalities [5]. The simulated interactions offer students a safe, low-risk environment in which they can practice conducting patient interviews, make mistakes, and receive direct feedback—all of which have been shown to significantly enhance clinical communication skills [6].

A fundamental element in constructing realistic patient simulations is the clinical vignette—a concise, precise description of a hypothetical scenario [7]. In medical education, vignettes are commonly used to represent complex clinical situations, assess diagnostic reasoning, and evaluate students’ decision-making [8]. For a vignette to serve as a solid foundation for patient simulation, it must encompass not only primary medical information, such as current symptoms and medical history, but also secondary factors that influence communication, including demographic details, health literacy, socioeconomic status, and personality traits [9].

Standardized patients have proven effective in teaching basic conversation techniques, but they have significant limitations. The availability and scalability of standardized patient programs are limited by high logistical and financial costs, standardization between different actors is difficult to ensure, and the authentic simulation of complex psychosocial contexts is only possible to a limited extent for logistical reasons (eg, foreign languages, involvement of children, or emotionally extremely stressful topics such as domestic violence). Technical simulation dolls are primarily suitable for training procedural skills but offer only limited possibilities for training complex communicative interactions that require emotional sensitivity and adaptive responses [10]. Digital technologies now offer promising alternatives. Recent advances in large language models (LLMs) have enabled the dynamic generation of text based on patterns learned from vast corpora, making it possible to simulate dialog-based patient interactions. Interaction with LLMs is facilitated through user and system prompts—carefully engineered instructions that ensure the model has all relevant information to perform the desired task accurately.

### Related Work and Contribution

The importance of psychosocial factors influencing doctor-patient communication has increased significantly in recent years. Factors such as migration background, language barriers, socioeconomic status, and cultural differences can hinder communication, promote misunderstandings, and impair therapy adherence and equal health opportunities [11,12]. Research on institutional mistrust, which can arise from historical discrimination and negative experiences and has a lasting impact on the quality of treatment, is particularly relevant [13]. Experienced discrimination in a medical context is directly linked to increased skepticism toward health care institutions and reduced use of care [14,15]. Dealing with these psychosocial factors during patient-doctor interactions becomes more important in medical education. This work aims to create an interactive tool that can be used to train communication and interaction skills in this context.

The potential of LLMs for the simulation of standardized patients has recently been demonstrated by multiple studies. In medical training, Cook et al [16] demonstrated the viability of using GPT-4 (OpenAI) for simulating standardized patients, evaluated based on anthropomorphism, clinical accuracy, and adaptability. The researchers also showed that the LLM can score the quality of the medical assessment. Similarly, Cook et al [16] compared 2 models (GPT-4.0-Turbo and GPT-3.5-Turbo [OpenAI]) for patient simulation and demonstrated their capability of simulating dialogues, representing patient preferences, and providing personalized feedback. In a second, preliminary study, Cook [17] also performed patient simulation and limited testing using Claude (Anthropic), performing “exceptionally well.” Öncü et al [18] applied GPT-4o (OpenAI) to create an environment for intern physicians to practice case-management skills. Borg et al [19] combined the LLM-based simulation (GPT-3.5-Turbo [OpenAI]) with social robotics, comparing this setup with a conventional, computer-based simulation for fostering clinical reasoning skills in medical students. The mixed methods study showed that 15 students perceived the LLM-enhanced variant as more authentic and providing a beneficial overall learning effect. Targeting nursing education, Benfatah et al [20] showed that a small sample of 12 nursing students embraced the LLM-based interaction and recognized its value in training.

LLMs have not only demonstrated their potential to simulate virtual patients but also seem capable of generating the underlying clinical vignettes efficiently. For example, Coskun et al [21] conducted a randomized, controlled experiment for vignette generation using ChatGPT-3.5 (OpenAI) and showed that the quality of vignettes is comparable to those created by human authors. Another 2 studies demonstrate the vignette generation capability of LLMs for Japanese specifically [22,23].

We can recognize that LLM-based vignette generation and virtual patient simulation have recently received increased attention in medical informatics research. However, previous research seems to focus on each task separately (ie, vignette generation, patient simulation). Furthermore, none of the studies mentioned above applied a user-centered development approach but rather performed experiments measuring the capability of the LLM-based simulation. Therefore, none of the studies focused on the integration of a simulation tool into existing learning processes of medical students. For example, Cook [17] used a simple, text-only Python interface.

We furthermore note that most of the studies were conducted using commercial models provided by OpenAI and do not include a comparison to other providers or models except Cook [17], who conducted limited testing with Claude (Anthropic).

### Goals of the Study

Existing LLM applications in the medical education context focus primarily on medical content or general communication scenarios and neglect the systematic integration of psychosocial contexts. With this paper, we want to introduce a digital tool that leverages LLMs to simulate patient conversations based on clinical case vignettes that can be used for medical education purposes. In contrast to other patient simulators, we want to integrate the psychosocial context and offer the opportunity to train communication with patients with varying psychosocial contexts. To support the generation of case vignettes, we propose a human-in-the-loop approach consisting of a comprehensive case template and a vignette generator. In this paper, we describe the user-centered design process, the technical implementation of the tool, and the first results from usability testing. Furthermore, our goal is to study differences of LLMs in simulating patients based on case vignettes in an exploratory study. The simulation tool focuses specifically on the process of collecting the medical history.

## Methods

### Requirement Collection

The system development followed principles of user-centered design. Medical students, who are the targeted user group, were involved in the requirement collection process and in the usability testing. Explorative model evaluation involved medical doctors.

The initial system requirements were identified through a literature analysis and qualitative interviews with medical students as prospective end users. A total of 2 third-year medical students participated in the interviews (2/9 contacted), recruited from the social network of the authors. Both were in the 6th semester of their studies of medicine. To accelerate data collection and focus on the discussion, early functional prototypes were presented to the participants as a basis for discussion. The requirements were qualitatively analyzed and prioritized based on their perceived relevance as indicated by the students, with specific focus on the usefulness for learning clinical communication skills. This process yielded the following key functional requirements:

1. Vignette management: the system must provide a wide range of patient cases, ideally sortable by medical specialty and year of study.
2. Organization: case vignettes should be organized using a folder system inspired by familiar learning platforms.
3. Artificial intelligence (AI)–based simulation: patient simulations should be powered by generative AI to enable unpredictable and realistic dialogues.
4. Interaction design: the simulated patient should not reveal information directly but instead respond only to targeted inquiries, encouraging natural conversation flow.

### Template for Case Vignettes

Building on the work of Reichenpfader and Denecke [9], a highly structured vignette template was developed to ensure realistic and diverse patient representations. Each vignette is technically stored as a JSON object within a JSONB field in the PostgreSQL (version 17; PostgreSQL Global Development Group) database. The structure is defined in the Python (version 3.11.9; Python Software Foundation) backend, which dynamically generates the user interface for vignette creation. The template comprises four main categories: (1) demographics, includes name, age, gender, occupation, and educational background; (2) medical history covers current symptoms, preexisting conditions, medications, allergies, and family history; (3) personality and communication describes health literacy, communication style, personality traits, and emotional state; and (4) social factors capture social support systems, socioeconomic status, cultural background, and language proficiency.

To facilitate thematic organization of the generated vignettes, the classification system of the Canadian Emergency Department Information System (CEDIS) Working Group [24] was implemented. Its suitability for use in medical education was confirmed by the participating students during the requirement analysis phase. Example vignettes can be found in Multimedia Appendix 1.

### System Architecture

#### Overview

**Figure 1:**
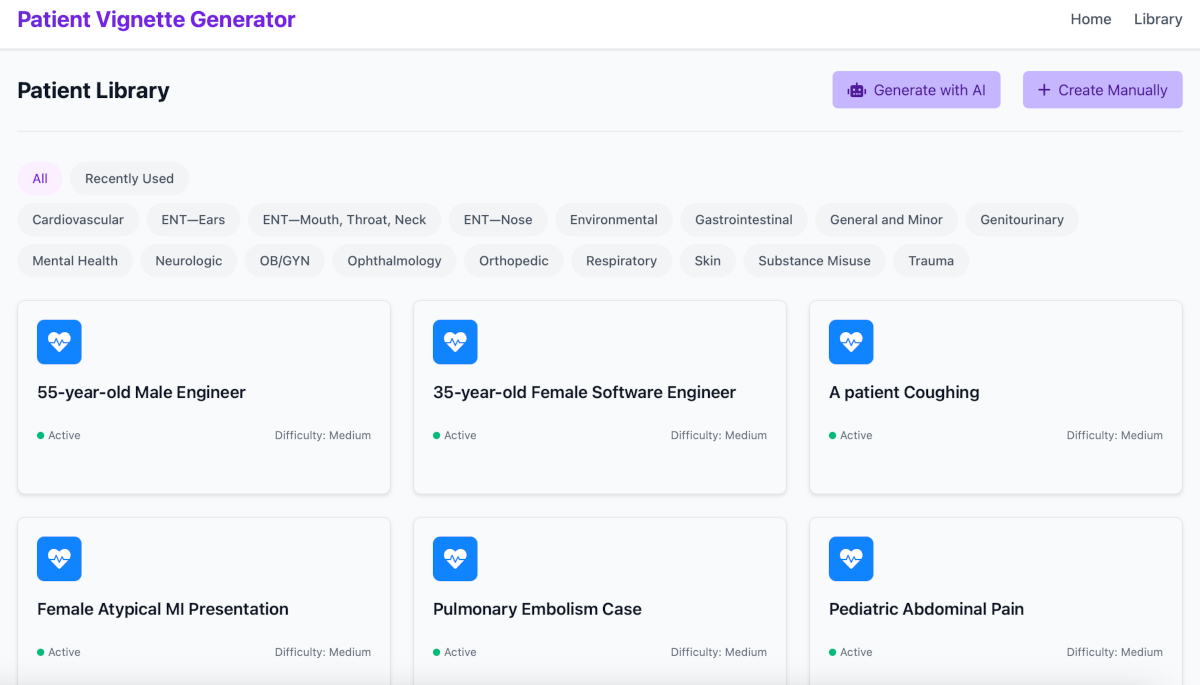
Library of case vignettes. Each vignette is represented by a rectangle. Filtering along Canadian Emergency Department Information System categories is possible.

**Figure 2:**
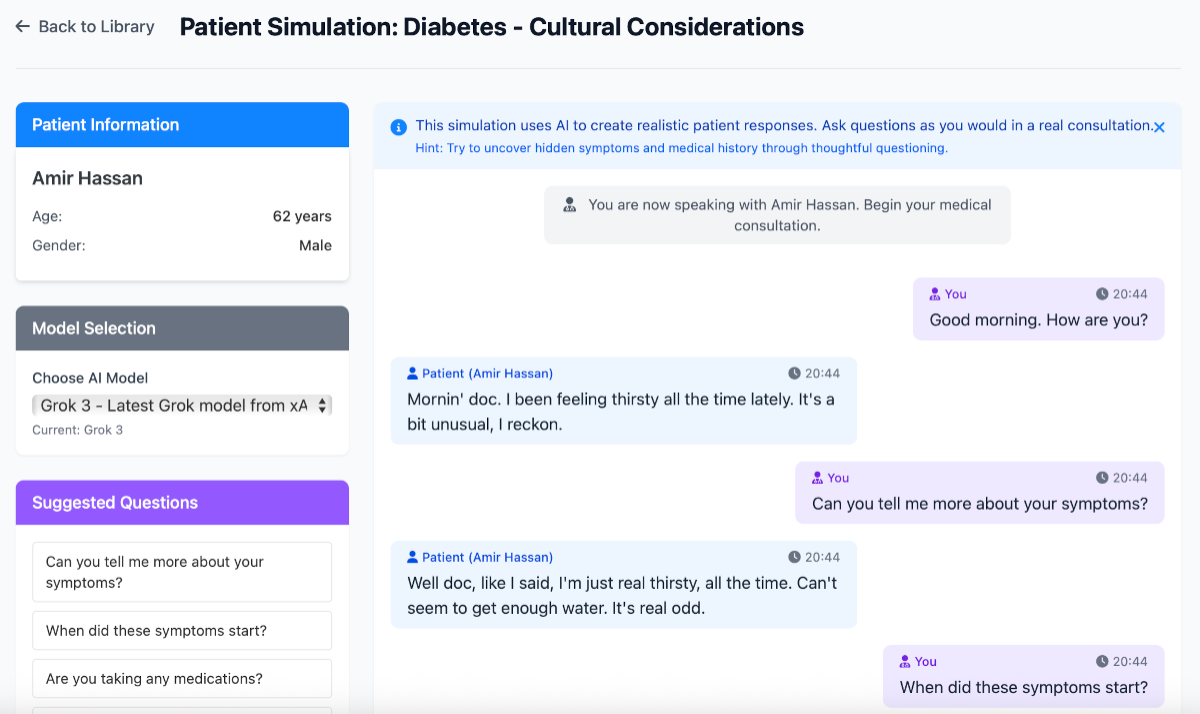
User interface for the interactive simulation tool. On the left, patient information is shown, together with the selected model and suggested questions as guidance for the interaction. On the right, the simulation chat is shown.

Our fully functional, web-based prototype for LLM-supported patient simulation includes the following core components: (1) AI-assisted case vignette generator—users can create vignettes manually by specifying values in a structured vignette form or have a complete vignette generated by interacting with an LLM using a “meta-prompt.” All vignettes can be organized in a folder structure (Figure 1). (2) Dynamic patient simulator—users can select a vignette and conduct a medical history gathering with a simulated patient (Figure 2).

Access to the current version of the prototype will be provided upon request.

**Figure 3:**
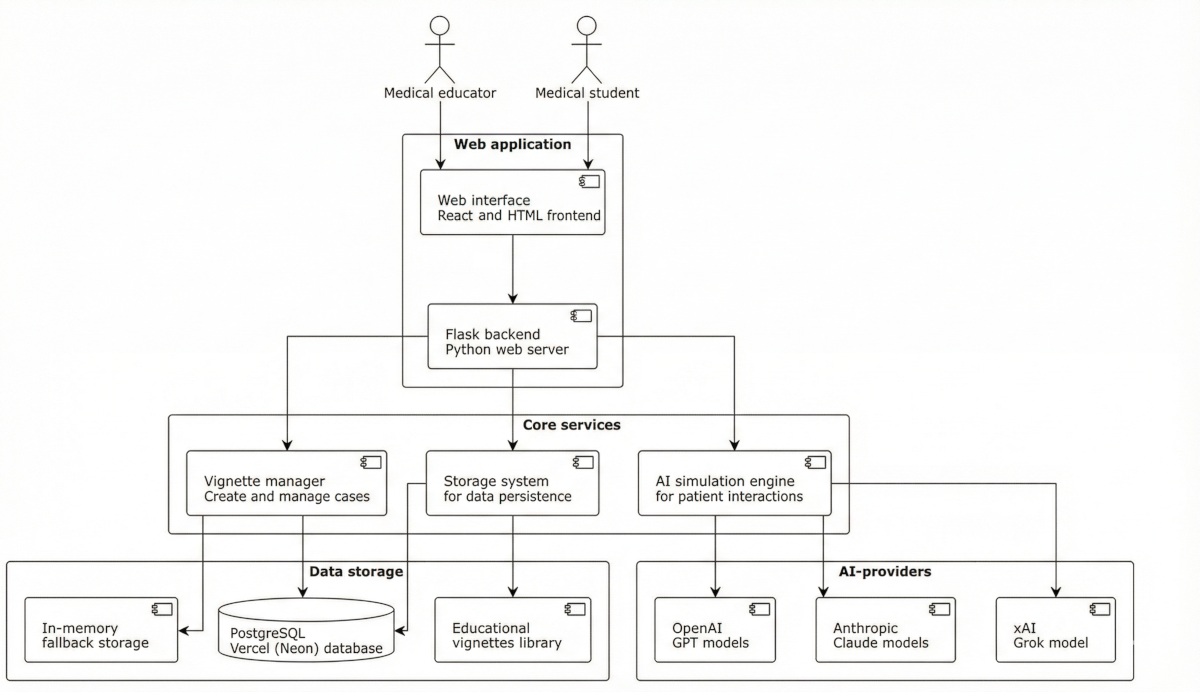
System architecture showing the users, the web application, the core components, data storage, and external artificial intelligence (AI) services.

The system is implemented using a multilevel client-server architecture (Figure 3). Users interact with a web application consisting of a reactive frontend and a backend built with Flask in Python. The backend orchestrates several core services—a vignette management system for handling case definitions, a storage layer for data persistence, and an AI simulation engine that encapsulates communication with external AI services (OpenAI, Anthropic, xAI). The following technologies were used for implementing the prototype:

1. Backend: Python (version 3.11.9) with the lightweight web framework Flask (version 3.0.2). Database access is abstracted via SQLAlchemy (version 2.0.41).
2. Database: PostgreSQL (version 17) was chosen for reliable and scalable data management.
3. Frontend: the user interface is implemented using standard web technologies (HTML5, CSS3).
4. Deployment: the application is deployed via the Vercel platform [25].

To ensure technological breadth and flexibility, the prototype developed in this work integrates 5 LLMs from 3 leading AI providers. These include GPT-4 and GPT-3.5-Turbo by OpenAI, Claude 3 Opus and Claude 3 Sonnet by Anthropic, and Grok 3 by xAI.

In the following, we provide more details on the two components.

#### AI-Assisted Case Vignette Generator

This component enables the generation of case vignettes using an LLM. It constructs a prompt that instructs an LLM to assume the role of a medical education expert and to output a case vignette in a predefined JSON format. The implementation incorporates multiple technical elements:

1. Strict output format: the prompt enforces a rigid JSON schema, including a naming convention of the form “[age]-year-old [gender] [occupation],” explicitly excluding any medical diagnoses.
2. Medical categorization: the LLM is provided with 17 predefined categories from the CEDIS. Internally, these are enriched with priority levels (CEDIS_PRIORITY) and icon mappings (CEDIS_ICONS) used for the visual representation of vignettes.
3. Clinical accuracy: the prompt includes explicit instructions to ensure realistic clinical content, such as appropriate medications, relevant comorbidities, and age-appropriate conditions.

The response from the LLM is processed by a parsing function, which uses boundary detection to extract the JSON object and validate the required fields. The generated tags are cross-checked against existing categories in the database. In case of generation failure, a fallback mechanism uses regex-based extraction to create a minimal vignette. An example LLM prompt with a generated case vignette is provided in Multimedia Appendix 2.

#### Dynamic Patient Simulator

The dynamic patient simulator produces highly personalized prompts for simulating realistic patient dialogues. At the core of the prompts is a strict role instruction, reinforced multiple times, ensuring that the LLM remains consistently in character as the patient: “You must stay in the character of the patient at all times. Never break character.”

A key mechanism for realistic medical history taking is gradual symptom disclosure. The prompt explicitly instructs the LLM to reveal information incrementally. This is technically implemented through the hierarchical structuring of symptoms into primary and secondary groups, where secondary information may only be disclosed upon several conversation turns.

Patient behavior is dynamically modeled based on case vignette data through the following mechanisms:

1. Language modeling: the language complexity is adapted to the patient’s health literacy and educational background. Patient simulations with lower literacy levels are instructed to use everyday language, analogies, and occasional grammatical errors, while those with higher competence may use appropriate medical terminology.
2. Personality modeling: 12 predefined personality traits (eg, anxious, stoic) are mapped to specific behavioral instructions. For example, an anxious patient is guided to frequently seek reassurance, while a stoic one is prompted to minimize or downplay symptoms. These traits may interact to produce nuanced behaviors.
3. Anxiety-level integration: a numeric anxiety score (1-5) is translated into detailed behavioral descriptions ranging from “very calm and composed” to “extremely anxious and distressed.”

To implement this behavioral control, advanced prompt engineering techniques are used, including negative instructions (eg, “NEVER reveal all symptoms at once”) and few-shot learning. The latter is realized through the inclusion of concrete dialogue examples within the prompt to guide the model toward the desired conversational style [26-28]. An example prompt generated from a case vignette is provided in Multimedia Appendix 1.

### System Evaluation

#### Formative Usability Test

To get feedback on the prototype and to identify aspects for improvement, we conducted a formative task-based usability test. It was conducted with 5 medical students; 1 student was in the 12th semester, 2 were in the 10th semester, and 2 were in the 6th semester. Participants were asked to complete 4 standardized tasks that cover the core functionalities of the application: manual vignette creation, AI-assisted vignette generation, organizing vignettes using folders, and conducting a patient simulation.

To quantitatively assess usability, the standardized System Usability Scale (SUS) was used [29]. Following the usability test, the participants were asked to answer a questionnaire with open questions about their user experience, specific strengths, weaknesses, and suggestions for improvement. These qualitative data were analyzed using a thematic analysis conducted by 1 coauthor.

#### Exploratory Language Model Comparison

Following the usability evaluation, a comparative analysis was conducted to assess the performance and differences of different LLMs in simulating patients based on case vignettes. The aim of this evaluation was to determine which model produces the most realistic and coherent patient simulations for medical history taking. A quantitative approach was applied. The participants consisted of 4 practicing physicians from the Department of Infectious Diseases at the University Hospital Basel, who served as domain experts. The group consists of 2 senior physicians and 2 assistant doctors, all aged 30 years or older, with an equal gender distribution (2 men and 2 women). Each expert conducted 3 simulated history-taking interviews using the same set of case vignettes, with each simulation powered by a different LLM. The evaluation was carried out using the developed web application. The evaluation cases were selected considering their clinical relevance to the participating physicians:

- Case 1: community-acquired pneumonia
- Case 2: complicated urinary tract infection
- Case 3: cellulitis in a diabetic patient

For the comparative evaluation of simulation quality, a targeted selection of models was made (GPT-4, Claude 3 Opus, and Grok 3). This selection represents a cross-section of state-of-the-art generative language models (in May 2025) and was intended to capture a range of capabilities in natural language understanding and dialogue generation relevant to realistic patient simulations.

A custom questionnaire was developed to rate the simulation quality across 7 criteria, using a 5-point Likert scale (ranging from “Strongly disagree” to “Strongly agree”). The evaluated criteria were (1) coherence of the symptom profile, (2) natural conversational flow, (3) patient-like language, (4) responsiveness to follow-up questions, (5) realistic emotional expression, (6) realistic uncertainty and conversational lapses, and (7) plausible time and symptom progression.

Participants received detailed written instructions. For each of the 3 cases, they were asked to launch the corresponding simulation, select the assigned LLM, and conduct a 2-5-minute history-taking interview in their preferred language. Immediately after each simulation, they completed the corresponding evaluation form. It is important to notice that each LLM was tested with a single case vignette, chosen to minimize expert workload. Neither the selected model nor the case was blinded to the participants.

Data were analyzed using Python 3 with SciPy and scikit-posthocs libraries. Normality was assessed using Shapiro-Wilk tests; given violations of normality assumptions (all *P*<.001), nonparametric procedures were used. The primary analysis used the Friedman test to compare ratings across the 3 models, accounting for the repeated-measures design. Post hoc pairwise comparisons used Wilcoxon signed-rank tests with Holm-Bonferroni sequential correction (*k*=3 comparisons, family-wise α=.05) and Cliff δ for pairwise comparisons. Interrater reliability was assessed using the Kendall coefficient of concordance (W).

### Ethical Considerations

All participants provided informed consent to take part in the study, agreeing to the anonymized analysis and publication of their responses. The study focused on quality assurance and usability evaluation of a patient simulator prototype and did not involve patient data or interventions. Accordingly, formal ethics committee approval was not required in accordance with institutional and national guidelines [30]. None of the participants received any compensation for their participation in the study.

## Results

### Results From Usability Testing

The analysis of the SUS assessment was performed using an online calculator. The individual SUS scores from the 5 participants were 82.5, 82.5, 95.0, 97.5, and 100. Based on these values, the mean SUS score was calculated as 91.5 (SD 8.4). According to established benchmarks, a SUS score above 90 is generally considered “excellent” usability, in our case, high perceived usability in a small formative sample [31]. A post hoc analysis of the sample size was conducted based on the observed variability in SUS scores. With 5 participants (mean SUS score 91.5, SD 8.40), the 95% CI for the mean SUS score was 81.1-101.9, corresponding to a margin of error of ±10.4 points. While the sample size offers limited precision, this degree of uncertainty is acceptable for a formative usability evaluation, the objective of which is to identify significant usability issues rather than to achieve high statistical accuracy.

The thematic analysis of the open questions revealed consistent subject areas. The simplicity and intuitive usability of the system were unanimously cited as key strengths. The participants described the navigation as clear and well-organized and positively emphasized the appealing, simple visual design. There was a consensus that interaction with the system could be learned very quickly and without significant training.

At the same time, clear potential for improvement was identified. The most frequently mentioned point of criticism was the lack of a formal conclusion after the simulation. The students wanted feedback on their interaction, which is currently not provided, and a summary of the case in order to maximize the learning effect. One tester, who is about to graduate, noted that “only the case history is not enough” for them to use the tool regularly and therefore rated the application as “rather not” helpful for their studies. Furthermore, ideas for future extensions were mentioned, such as a note field or the integration of laboratory values.

### Statistical Analysis of Model Comparison

#### Experimental Design

A within-participants experimental design was used to evaluate the performance of 3 LLMs (Grok 3, GPT-4, and Claude 3 Opus). Four independent raters (R1-R4, health professionals) assessed each model across 3 distinct use cases, answering 7 evaluation questions (Q1-Q7) for each case. Each rater evaluated all 3 models, with one model assigned to each case. This resulted in a balanced design with 84 total observations (N=84). Responses were recorded on a 5-point Likert scale (1=lowest; 5=highest), though the observed range was restricted to 2-5, with no ratings of 1 recorded.

#### Descriptive Statistics

**Figure 4:**
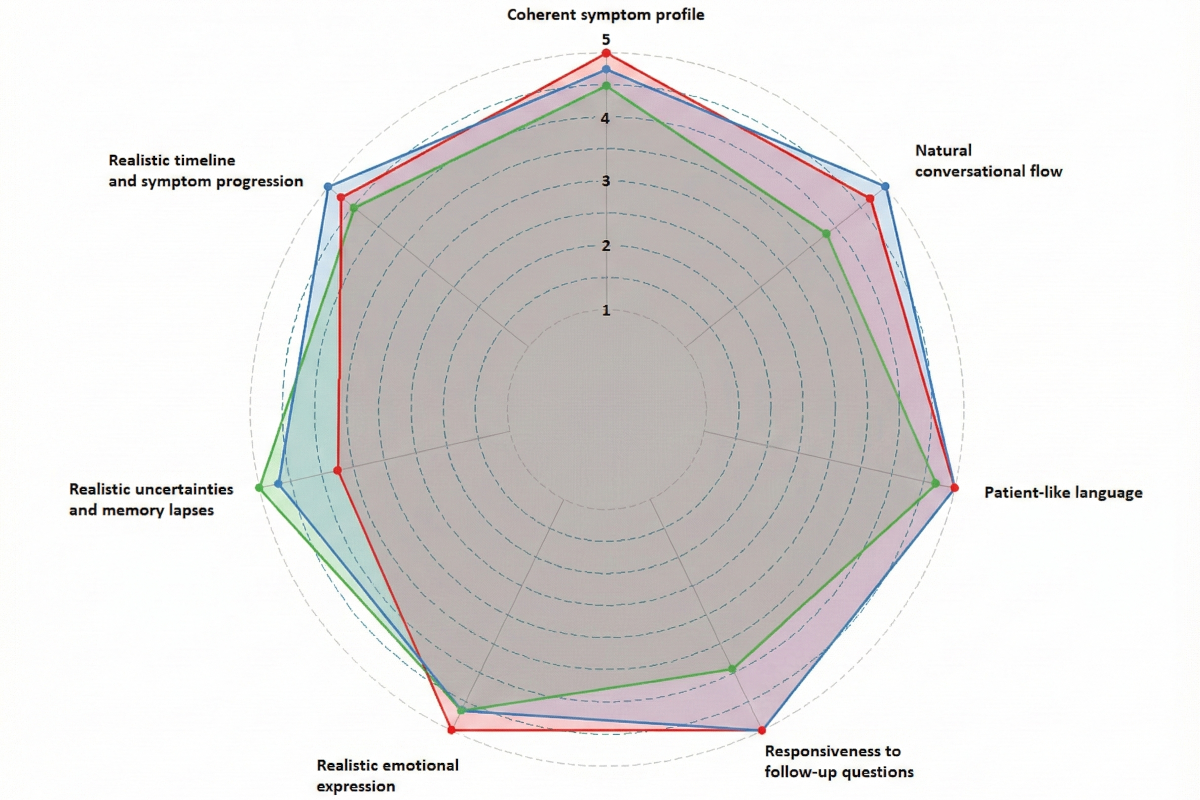
Strengths and weaknesses of 3 large language models (LLMs) for simulating patients. Colors refer to the different LLMs: green=Grok 3, red=GPT-4, and blue=Claude 3 Opus.

The quantitative evaluation by 4 health professionals provided the differences in the LLM results as shown in Table 1 and Figure 4. Ratings showed limited discriminative reliability with an intraclass correlation coefficient (2, 1) of 0.165, and a pronounced ceiling effect, with most scores clustered between 4 and 5, constraining interpretation. Accordingly, all group differences should therefore be viewed as exploratory. For the 3 criteria, bigger differences in ratings can be recognized. We can see that Grok 3 and Claude 3 Opus performed better regarding realistic uncertainties and memory lapses than GPT-4. Regarding responsiveness to follow-up questions and natural conversation flow, Grok 3 was less good than the other two LLMs.

**Table 1:** Comparison of language models’ quality assessed by clinical experts (n=4; scale 1 “totally disagree” to 5 “totally agree”).

Evaluation criterion	Grok 3, mean (minimum-maximum)	GPT-4, mean (minimum-maximum)	Claude 3 Opus, mean (minimum-maximum)
The symptom profile described by the simulated patient is coherent.	4.5 (4-5)	4.75 (4-5)	4.25 (3-5)
The simulated patient describes the symptom presentation in language typical of real patients.	4.25 (4-5)	4.25 (4-5)	4.0 (2-5)
The simulated patient can describe realistic timelines and symptom progression.	4.75 (4-5)	4.5 (4-5)	4.25 (4-5)
The simulated patient displays realistic uncertainties and memory lapses.	3.75 (3-5)	3.0 (2-4)	4.0 (3-5)
The emotions portrayed appear realistic in the context of the simulated situation (medical history taking).	3.5 (3-4)	3.75 (3-4)	3.5 (2-5)
The flow and pace of the conversation appear natural.	4.25 (3-5)	4.0 (3-5)	3.25 (2-4)
The simulated patient responds appropriately to targeted follow-up questions.	4.75 (4-5)	4.75 (4-5)	3.75 (2-5)

Grok 3 demonstrated strengths in depicting realistic timelines and symptom progression, as well as in providing appropriate responses to follow-up questions (both mean scores 4.75, SD 0.5). Its lowest score was observed in the portrayal of realistic emotions (mean 3.5, SD 0.58).

GPT-4’s primary strength was the coherence of the symptom presentation (mean 4.75, SD 0.5), outperforming the other 2 models in this category. It also matched Grok 3 in its ability to respond appropriately to follow-up questions (mean 4.75, SD 0.5). However, GPT-4 showed the weakest performance in conveying realistic uncertainties and memory lapses (mean 3.0, SD 0.82).

Claude 3 Opus exhibited the greatest variance in its ratings. The lowest scores of this model were found in the categories of natural conversation flow (mean 3.25, SD 0.96) and the portrayal of realistic emotions (mean 3.5, SD 1.0). Notably, the use of language typical of real patients (mean 4, SD 1.41) and the ability to respond appropriately to follow-up questions (mean 3.75, SD 1.26) showed the highest variability in responses. Figure 4 shows the comparison of the 7 evaluation criteria and the 3 language models in a spider chart.

A Friedman test was conducted to evaluate differences in ratings across repeated measures (raters and questions) among the 3 models (Table 2). None achieved statistical significance at the α=.05 level (all *P* values of >.14). The analysis revealed no statistically significant differences in performance between the models (*χ*²_2_=2.086; *P*=.45). Kendall coefficients of concordance (W values) are small (≈0-0.19), and the CIs are wide, so the degree of agreement on model rankings is very uncertain, suggesting that there are minimal practical differences in the performance of the models.

**Table 2:** Model comparison using Friedman tests per question: chi-square value, *P* value, and Kendall coefficient of concordance (W) with a bootstrap 95% CI to indicate agreement on model ranking.

Question	Chi-square (*df*)	*P* value	Kendall W (95% CI)
Q1	3.00 (2)	.22	0.14 (0.00-0.61)
Q2	0.00 (2)	>.99	0.00 (0.00-0.42)
Q3	3.00 (2)	.22	0.14 (0.00-0.61)
Q4	3.00 (2)	.22	0.14 (0.00-0.61)
Q5	0.67 (2)	.72	0.05 (0.00-0.61)
Q6	0.93 (2)	.63	0.11 (0.00-0.81)
Q7	4.00 (2)	.14	0.19 (0.00-0.75)

#### Interrater Comparison

Examination of rater-specific statistics revealed considerable heterogeneity in rating patterns. R1 exhibited the most lenient rating tendency (mean 4.524, SD 0.794), while R2 demonstrated the strictest ratings (mean 3.762, SD 1.064), exhibiting the greatest variability. Notably, R2’s mean rating for Claude 3 Opus (mean 2.714, SD 0.881) was substantially lower than the ratings given by the other evaluators, which suggests either rater bias or a divergent interpretation of the evaluation criteria. R4 displayed the most consistent rating pattern, only rating with 3 or 4; however, this consistency may reflect restricted range use rather than genuine reliability.

Interrater reliability was assessed using the Kendall coefficient of concordance (W) across raters. Specifically, we thereby assessed how consistently they rank the 3 models. We used a bootstrap over raters (resampling columns with replacement) for percentile 95% CIs. Results are shown in Table 3. Values for Kendall W are all very low (0-0.19), showing at best a weak agreement. CIs start at 0 and extend up to 0.6, reflecting extreme imprecision.

**Table 3:** Interrater agreement per question, reported as the Kendall coefficient of concordance (W) across raters.

Question	Kendall W (95% CI)
Q1	0.141 (0-0.609)
Q2	0.000 (0-0.422)
Q3	0.141 (0-0.609)
Q4	0.141 (0-0.609)
Q5	0.047 (0-0.609)
Q6	0.109 (0-0.812)
Q7	0.188 (0-0.750)

#### Effect Size Analysis

Table 4 presents the results of the post hoc pairwise comparisons. We treated the data as within-rater and used a cluster bootstrap over raters to get a 95% CI percentile for Cliff δ. We calculated Cliff δ pooled across all 7 questions. None of the 3 pairwise comparisons achieved statistical significance after Holm-Bonferroni correction. The comparison between Claude 3 Opus and Grok 3 approached but did not reach significance (W=30.00; *P*=.07; Holm α=0.017), with a medium effect size (*r*=0.322). The comparison between Claude 3 Opus and GPT-4 showed a small effect (W=31.50; *P*=.18; *r*=0.249), while the comparison between GPT-4 and Grok 3 demonstrated a negligible effect (W=22.50; *P*=.59; *r*=0.096). In practice, the signed-rank statistics are very small because there are only 4 paired observations per contrast. With 4 raters, there is no robust evidence that any model consistently outperforms another on any single criterion. The data are descriptive rather than confirmatory.

**Table 4:** Results for post hoc Wilcoxon signed-rank tests, Holm-corrected for multiple comparisons.

Comparison	n	W statistic	*P* value	*z* score	Effect size (*r)*	Mean difference	Holm α	Cliff’s δ (95% CI)
Claude 3 Opus vs Grok 3	28	30	.07	–1.704	0.322	–0.393	0.017	–0.21 (–0.591 to 0.059)
Claude 3 Opus vs GPT-4	28	31.5	.18	–1.318	0.249	–0.286	0.025	–0.15 (–0.128 to 0.556)
GPT-4 vs Grok 3	28	22.5	.59	–0.509	0.096	–0.108	0.05	–0.07 (–0.184 to 0.128)

## Discussion

### Principal Findings

This paper presents a training system for medical students to train interaction with diverse patient populations using LLMs and generating vignettes for case-based learning. The evaluation shows good usability and provides first insights into the differences of LLMs when simulating patients and their characteristics.

### Usability and User Experience

The SUS results from 5 participants provide an initial indication that the tool may fulfill its core requirements for simplicity, efficiency, and appeal. The technical implementation can thus be deemed a success and forms a robust foundation for further development. However, good usability alone does not suffice to create an effective medical learning instrument. Qualitative responses highlighted a crucial shortcoming, that is, the repeated wish for a “didactic loop”—a cycle of history taking, diagnostic feedback, and case summary—was identified as an important finding in the qualitative evaluation. Without this step, the application remains primarily a tool for communication training, leaving its full potential for fostering clinical reasoning and diagnostic skills untapped. Such a feedback mechanism is considered critical for sustainable learning and repeated use. Traditionally, simulated patients are not only tasked with portraying a case but also with providing structured feedback to learners. This complex requirement—involving both role-play and performance evaluation—necessitates extensions to our simulator. An additional extension would be to consider the guidelines and communication strategies taught in medical education and to assess whether the students apply them in their interactions.

The feasibility and validity of an AI-based feedback system were demonstrated by Holderried et al [32], who showed that a GPT-4–powered chatbot could generate patient simulations and provide automated feedback, which closely matched expert human evaluations (Cohen κ=0.832). Their approach—using a dedicated “feedback prompt” to analyze the dialogue—directly aligns with the development pathway proposed in this work. This feedback capability of LLMs was also demonstrated by Cook et al [16] using GPT-3.5-Turbo.

### Quality of AI-Generated Simulations and LLM Comparison

This analysis provides no evidence for statistically significant differences in performance among the 3 evaluated LLMs. Our findings should be interpreted in light of the limited discriminative signal in the rating data, reflected by very low interrater reliability and a strong ceiling effect (67/84, 81% of all ratings at 4-5). This reflects clearly the small rater panel. Accordingly, the comparative results are exploratory and underpowered and are offered as preliminary indications rather than definitive effects.

Overall, results show the general suitability of the tool, as all tested models were able to generate plausible, realistic patient roles useful for practicing history taking, with each statement rated between “Neutral” (3 points) and “Strongly agree” (5 points), and no ratings of “Strongly disagree” (1 point). These findings echo previous studies, which have also found that current LLMs can create clinical vignettes with high linguistic and medical accuracy, though expert revision is often required. For instance, Yanagita et al [23] found 97% of LLM-generated vignettes usable with minor modifications, while Takahashi et al [22] noted considerable potential for ChatGPT-4–generated cases in medical education, but with room for improvement in realism. Our system differs from such approaches, as we are suggesting a human-in-the-loop approach for vignette generation, providing 2 options—specifying all characteristics manually using the vignette template or using AI-support.

All models scored highest for symptom coherence and realistic timelines. Statements assessing the realism of the dialogue itself, however, received lower scores. This suggests that the models were better at maintaining factual accuracy than at simulating natural role play. The most significant difference between Grok 3 and GPT-4 was in their portrayal of realistic uncertainty (Figure 4). Although GPT-4 performed strongly overall and achieved the highest scores for symptom coherence and emotional realism, it struggled to depict realistic uncertainty—a key component of authentic medical interviews. By contrast, Claude 3 Opus received the lowest scores in most categories but performed best in conveying uncertainty. Grok 3 performed particularly well in core medical content, coherent symptom simulation, and realistic timeline generation. GPT-4 and Grok 3 demonstrated similar abilities when addressing follow-up questions, whereas Claude 3 Opus fell behind, particularly in terms of maintaining conversational flow and using language typical of patients. Claude 3 Opus also exhibited the greatest variability in expert ratings, particularly with regard to patient language, follow-up responses, and emotional realism. Furthermore, it was the only model to insert stage directions into the dialogue, which some users found distracting while others valued the added nonverbal context. These differences have to be considered with care, as each model was tested with a different case example, and the differences may also be due to the different sociodemographic characteristics of the simulated patients. Because of the small expert panel to judge the models, we cannot recommend any model as the primary model for medical patient simulations. Evaluations involving a larger number of raters are required to confirm these initial findings. Future improvements should also focus on prompting the models to simulate authentic human emotional expression and memory lapses, as these aspects are essential for the realism of clinical interviews.

In this study, we did not assess whether the answers from the simulated patient are realistic. A previous study showed that the answers of the simulated patient are correct, but the study was limited in size [9]. In principle, having an incomplete or even wrong answer from a simulated patient could be considered a realistic case, as in the real world, patients will not disclose everything and can share wrong information as well. This adds an additional dimension to the training scenario. In future work, we will study whether the correctness can be ensured and whether specific learning cases can be developed that allow training skills of diagnosing and medical history taking when patients are not honest or are incomplete in their answers.

### Educational Use Cases and Implications

As exemplified, we describe 2 educational use cases of the patient simulator. The first use case concerns the dynamic and authentic patient simulation integrating psychosocial complexity. In this use case, the simulator supports learning the patient-doctor interaction, given the psychosocial complexity of patients. Besides medical symptoms, the simulator uses a variety of contextual factors in the simulated interaction, including language barriers, family-related stressors, cultural sensitivities, and varying levels of trust or mistrust in the health care system. By integrating these dimensions, learners can experience and learn to overcome communication difficulties that often arise when treating diverse patient populations. For example, a patient with limited health literacy may misinterpret medical terminology, while a patient from a different cultural background may express distress indirectly or be reluctant to share personal information due to systemic mistrust.

A second use case focuses on training young surgeons to hold informed consent discussions with patients before surgical procedures. Such discussions are crucial for ensuring ethical and legally sound medical practice, as well as fostering patient trust and facilitating shared decision-making. However, novice clinicians often feel uncertain about communicating complex medical information, addressing patient fears, or managing emotional reactions under time pressure. For this use case, the simulator provides an authentic, dialogic environment in which students or young surgeons can practice and refine their communication strategies during simulated preoperative consultations. The AI-driven patient responds dynamically based on the learner’s explanations, empathy, and ability to balance technical accuracy with clarity and reassurance. This approach aligns with the principles of deliberate practice and experiential learning, emphasizing repeated, feedback-oriented engagement with realistic scenarios to achieve competence.

By interacting with the AI-based patient simulator, students will acquire domain-specific communication skills and develop a deeper understanding of the opportunities and limitations of AI in professional contexts. The simulated encounters provide an environment in which learners must navigate complex psychosocial dynamics, such as language barriers, family stressors, and mistrust of the health care system, requiring a high degree of empathy, cultural sensitivity, and adaptive communication strategies. Although promising, the use of a simulator within student education raises some ethical challenges. Among them is a risk for overreliance on scripted behavior, as LLM behavior might be predictable, and students miss learning to deal with unpredictable behavior. Biases can be amplified when the personality modeling is not diverse. Further, students need to understand that the simulator is a training aid, not a real patient interaction.

For educators, the system opens up new possibilities for integrating innovative AI tools into medical education in a meaningful way from a pedagogical perspective. It supports the expansion of teaching practice through technology-enhanced learning approaches, enabling instructors to design more personalized, reflective, and data-driven learning experiences.

At a broader societal level, the use of such a simulator addresses the urgent need to improve health care by facilitating more effective communication between physicians and patients from diverse backgrounds. By explicitly incorporating psychosocial factors such as language barriers, cultural differences, and institutional mistrust, the system helps to foster equity and inclusion in medical education, and ultimately in patient care.

### Limitations

Several methodological limitations must be considered when interpreting the results. The LLM evaluation was conducted with only 4 infectious disease experts, with low interrater reliability, limiting the statistical generalizability of the findings. In addition to these constraints, the study is statistically underpowered for detecting small or moderate differences between models. We used a frequentist analytic framework to generate CIs for the expert ratings; however, given the limited sample size, these intervals should be understood as descriptive indicators of uncertainty rather than tools for confirmatory inference. In other words, the frequentist estimates do not support strong population-level claims but instead provide a bounded range of plausible effect sizes based on the observed data. This aligns with the formative nature of the study. The statistical outputs offer preliminary signals to guide future, adequately powered evaluations rather than serving as evidence of definitive model differences. Because each model was tested on a single vignette and models were not counterbalanced across cases, the observed differences may also be due to the cases rather than model performance.

Only 2 students in their 6th semester contributed to the requirement collection process. Although attempted, it was not possible to gain the interest of additional students for this phase. The requirements were extended by information from scientific literature. It would be beneficial to get additional input from medical educators. Similarly, usability testing involved only 5 medical students, excluding the perspective of teachers in medical education who would use the tool for creating vignettes for teaching purposes. The raters were not specifically trained, which could have led to varying interpretations of the items of the Likert scale.

Each LLM was tested with a single case vignette, chosen to minimize expert workload but potentially limiting the assessment’s breadth. The analysis was also confined to 3 proprietary, closed-source models, without including open-source alternatives—reflecting a gap that future research should address. Further, the case and LLM were not blinded to the expert raters. This may have introduced bias, as they were able to see the name of the model they were interacting with.

The survey instrument used for expert evaluation was newly developed for this study and had not been previously validated or piloted, representing a challenge common to this research field, as described by Yanagita et al [23]. They therefore analyzed subjective expert opinions descriptively without mean comparisons. The usability test focused on learners’ perspectives without structured instructional units.

Subsequent studies should include a comprehensive evaluation with medical educators to validate the system’s suitability as a patient simulation tool. Larger numbers of raters and randomized model assignments to different vignettes would address current methodological limitations. A longitudinal study measuring actual learning outcomes in students who regularly train with the system, compared to a control group, would be necessary to empirically demonstrate didactic effectiveness.

### Future Work

In summary, this work successfully addresses the challenge of developing a highly usable simulation frontend and demonstrates the potential of LLMs as patient simulation tools. At the same time, it clearly outlines the necessary steps required to transform the tool into a comprehensive educational instrument. The most urgent improvement is the integration of an automated feedback mechanism that analyzes the conducted conversation and provides feedback to the student on what to improve. Building on validated approaches, another AI agent could analyze the dialogue and provide structured feedback on history-taking categories. This would address students’ expressed need for a formal case closure and clearer learning outcomes. The addition of digital note-taking tools and integration of structured data (eg, laboratory values, vital signs) would increase both complexity and realism, better meeting the needs of advanced learners. To have a more realistic training situation, the simulation tool should be extended by a voice user interface that allows a speech-based interaction.

Emerging LLMs such as GPT-4o, Gemini, Grok 3, and Claude 3.5 Sonnet now offer advanced multimodal capabilities. Future iterations could leverage these features to enrich vignettes with relevant visual findings, further enhancing didactic value.

## Appendix

Multimedia Appendix 1 Example prompts and vignettes.

Multimedia Appendix 2 Generation of a structured case vignette based on a prompt.

## Abbreviations

AI: artificial intelligence
CEDIS: Canadian Emergency Department Information System
LLM: large language model
SUS: System Usability Scale

## Data Availability

Access to the system can be provided upon request. Example prompts are available as multimedia appendices. Additional data sharing is not applicable to this article as no data sets were generated or analyzed during this study.
